# An annotated cDNA library of juvenile *Euprymna scolopes *with and without colonization by the symbiont *Vibrio fischeri*

**DOI:** 10.1186/1471-2164-7-154

**Published:** 2006-06-16

**Authors:** Carlene K Chun, Todd E Scheetz, Maria de Fatima Bonaldo, Bartley Brown, Anik Clemens, Wendy J Crookes-Goodson, Keith Crouch, Tad DeMartini, Mari Eyestone, Michael S Goodson, Bernadette Janssens, Jennifer L Kimbell, Tanya A Koropatnick, Tamara Kucaba, Christina Smith, Jennifer J Stewart, Deyan Tong, Joshua V Troll, Sarahrose Webster, Jane Winhall-Rice, Cory Yap, Thomas L Casavant, Margaret J McFall-Ngai, M Bento Soares

**Affiliations:** 1 Department of Medical Microbiology and Immunology, University of Wisconsin-Madison, Madison, WI, 53706, USA; 2 Department of Ophthalmology and Visual Science, University of Iowa, Iowa City, IA 52242, USA; 3 Department of Biomedical Engineering, University of Iowa, Iowa City, IA 52242, USA; 4 Department of Pediatrics, University of Iowa, Iowa City, IA 52242, USA; 5 Department of Electrical and Computer Engineering, University of Iowa, Iowa City, IA 52242, USA; 6 Department of Biochemistry, University of Iowa, Iowa City, IA 52242, USA; 7 Department of Orthopaedics, University of Iowa, Iowa City, IA 52242, USA; 8 Physiology and Biophysics, University of Iowa, Iowa City, IA 52242, USA; 9 Children's Memorial Research Center, Northwestern University, Chicago, IL, 60614, USA; 10 Pacific Biomedical Research Center, Kewalo Marine Laboratory, University of Hawaii, Honolulu, HI, 96813, USA

## Abstract

**Background:**

Biologists are becoming increasingly aware that the interaction of animals, including humans, with their coevolved bacterial partners is essential for health. This growing awareness has been a driving force for the development of models for the study of beneficial animal-bacterial interactions. In the squid-vibrio model, symbiotic *Vibrio fischeri *induce dramatic developmental changes in the light organ of host *Euprymna scolopes *over the first hours to days of their partnership. We report here the creation of a juvenile light-organ specific EST database.

**Results:**

We generated eleven cDNA libraries from the light organ of *E. scolopes *at developmentally significant time points with and without colonization by *V. fischeri*. Single pass 3' sequencing efforts generated 42,564 expressed sequence tags (ESTs) of which 35,421 passed our quality criteria and were then clustered via the UIcluster program into 13,962 nonredundant sequences. The cDNA clones representing these nonredundant sequences were sequenced from the 5' end of the vector and 58% of these resulting sequences overlapped significantly with the associated 3' sequence to generate 8,067 contigs with an average sequence length of 1,065 bp. All sequences were annotated with BLASTX (E-value < -03) and Gene Ontology (GO).

**Conclusion:**

Both the number of ESTs generated from each library and GO categorizations are reflective of the activity state of the light organ during these early stages of symbiosis. Future analyses of the sequences identified in these libraries promise to provide valuable information not only about pathways involved in colonization and early development of the squid light organ, but also about pathways conserved in response to bacterial colonization across the animal kingdom.

## Background

The study of the interactions of animals with their beneficial or mutualistic bacterial partners is a frontier field with a vast array of largely unanswered questions. A major subset of such associations, *e.g.*, those of mammals, comprises the horizontally transmitted partnerships, *i.e*., associations in which the host and symbiont population or community re-establishes the relationship with each new host generation [1]. For this specific array of partnerships, some principal questions include: how are the bacterial partners harvested from the environment? In associations with consortia of bacteria, who are the naturally coevolved symbionts, or residents, and who are the 'tourists'? What factors mediate partner recognition and specificity? How do the partners impact one anothers' developmental programs? How is stability of the association achieved so that the bacterial partners are not eliminated nor are they allowed to overgrow host tissues? What are the principal similarities and differences in the mechanisms underlying beneficial and pathogenic interactions?

Biologists know little about beneficial animal-bacterial relationships principally because of technological impediments, many of which have been alleviated by advances in molecular biology and biotechnology. These technological breakthroughs have poised the community of biologists to address the above-mentioned questions. Specifically, large-scale sequencing efforts are allowing biologists to define the communities associated with particular animal species (*e.g.*, [2-6]), and development of such methods as microarray analyses are enabling biologists to determine host and symbiont responses under natural and experimental conditions [6,7].

In addition to these technological advances, the development of powerful models for the study of animal-bacterial interactions has been essential. As in developmental biology, the study of a wide variety of models will shed light on evolutionarily conserved mechanisms as well as the basis of diversity. To this end, two principal, complementary model types are under investigation: those using germ-free or gnotobiotic animals and binary associations. Most notably for the former, Gordon and coworkers have been experimentally manipulating germ-free, gnotobiotic and 'conventionalized' (provided with their normal microbiota) zebrafish and mice to characterize interactions with their microbial partners [6,8]. These studies have revealed that the diverse community of microbiota in the vertebrate gut has profound effects on the gene expression, anatomy and physiology of the host animals (see *e.g.*, [9,10]). Binary associations, *i.e*., those composed on one host species and a population of one microbial species, occur most often in invertebrate animals and offer relatively simple experimental systems for the study of animal-microbe relationships [11-13].

**Figure 1:**
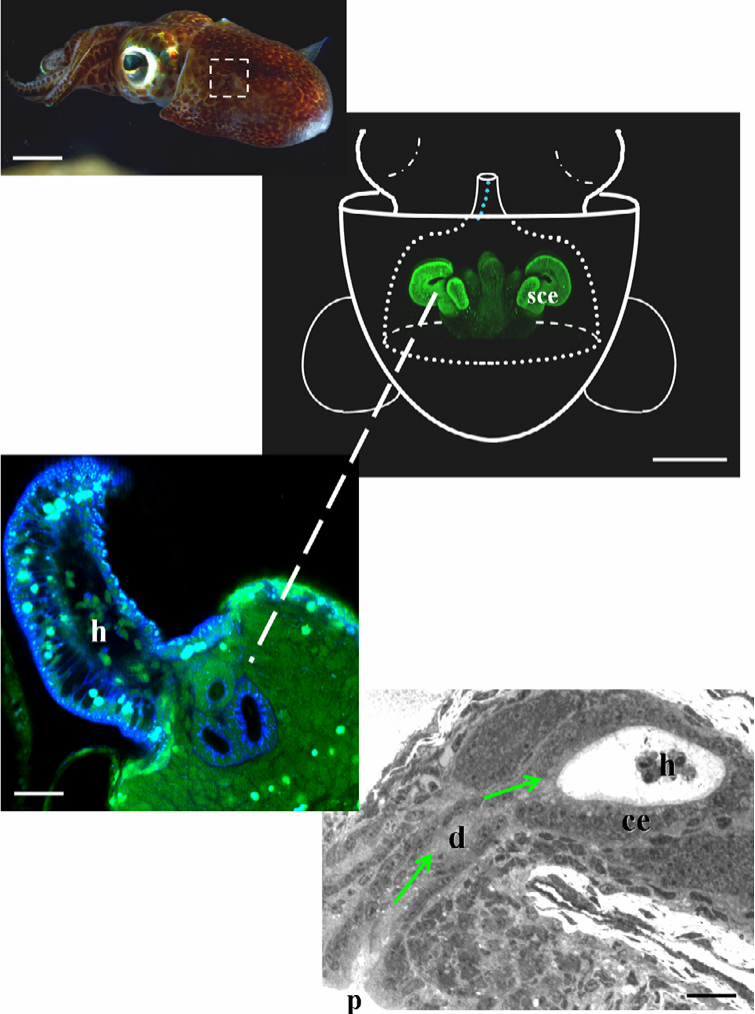
**The light organ system of *E. scolopes***. A. A swimming adult animal. The light organ (internal to the boxed area) is located in the center of the mantle cavity. Bar, 1 cm. B. A confocal image of a light organ (labeled with CellTracker, Molecular Probes) within a diagram of a newly hatched animal. The juvenile organ has a complex, superficial ciliated epithelium (sce) that facilitates colonization by the symbioint. Bar, 200 microns. C. A confocal image of the sites of *V. fischeri *entry into host tissues (labeled with acridine organ, AO). A set of three pores, into which aggregated *V. fischeri *cells will migrate, are located at the base of each lateral field of ciliated epithelial cells. In response to interactions with colonizing *V. fischeri*, host hemocytes (h) migrate into the sce, which will be lost during symbiont-induced light organ morphogenesis. Also visible in this image is the condensed chromatin characteristic of symbiont-induced apoptosis (arrows), which stains vividly with AO. Bar, 20 microns. D. A histological section revealing the path traversed by colonizing symbionts. Once aggregated in mucus outside the light organ, the symbionts enter the pores (p), travel up long ciliated ducts (d) and enter the crypt spaces where they interact with two cell types, the polarized epithelium that lines the crypts (ce) and a transient population of host hemocytes (h). Bar, 30 microns.

**Figure 2:**
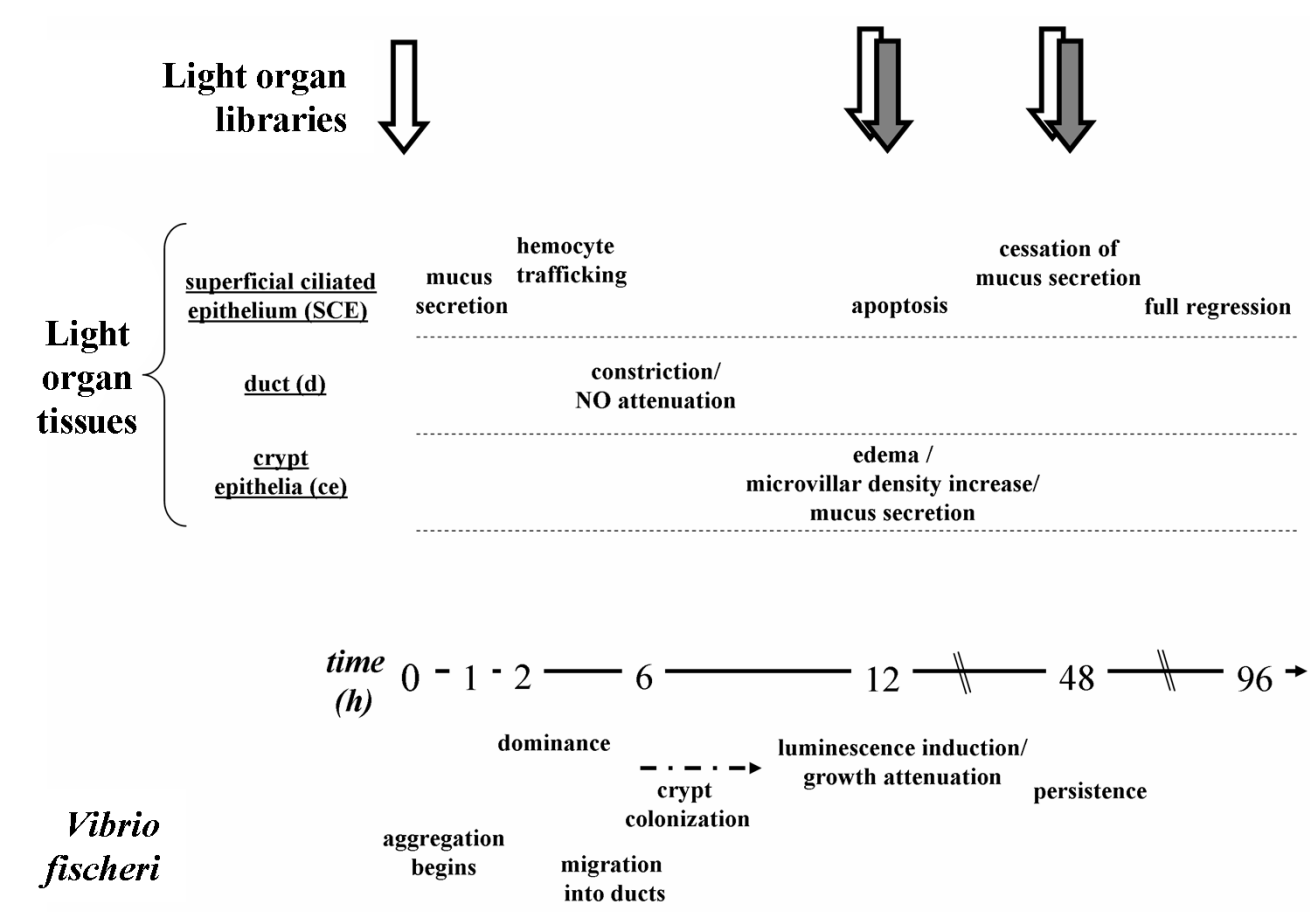
**The time points chosen for cDNA library construction**. The diagram illustrates when, in the context of the symbiont-induced developmental program of the host light organ and major milestones in *V. fischeri *colonization, tissues for library construction were dissected from juvenile animals. As the symbionts aggregate, migrate into host tissues and take up residence in the light organ crypts, they induce the diagrammed series of developmental changes in the various components of the organ. Most of these changes, including the irreversible loss of the SCE, which doesn't culminate until 96 hours, are triggered by or around 12 hours following first exposure to environmental *V. fischeri*. In addition, around 12 hours, the symbionts usually fully colonize the crypt spaces and their growth is attenuated and luminescence induced. By 48 hours, significant changes in the proteome occur (Lemus and McFall-Ngai, 2000). In addition, mutants of *V. fischeri *incapable of persisting in the light organ lose the ability to colonize the organ beginning at around 48 hours.

The association between the Hawaiian sepiolid squid *Euprymna scolopes *and its luminous bacterial partner *Vibrio fischeri *is a binary model system that has been under investigation for the last fifteen years (for review see [13]). The relationship, in which the symbiont lives extracellularly in crypts of the light organ, begins in the hours after the juvenile host hatches from the egg. During embryogenesis, the host develops a nascent light organ that promotes specific colonization by *V. fischeri *(Fig. 1). Once light organ tissues have been colonized, development of the partners is induced (Fig. 2). The bacterial symbiont triggers an elaborate developmental program in the host animal that leads to the transformation of the organ from an anatomy, physiology and biochemistry that promotes infection to one that fosters the functioning of the mature, bioluminescent organ. Likewise, the bacterial partner transforms its biology from that characteristic of its niche in the bacterioplankton to that of its niche as a luminous bacterial symbiont.

In developing the squid-vibrio model, researchers in the field have sought not only to describe the nature of the system, from both the host and symbiont sides of the association, but also to apply new tools, as they became available, for its study. Specifically, early efforts resulted in the development of genetics in the bacterial symbiont [14] and, more recently, the annotated sequence of the *V. fischeri *genome has become available [15]. The collaborative work presented here describes the first efforts to generate bioinformatics tools for the host side of the squid-vibrio association. Our goal was to define a representative set of host genes expressed in the juvenile light organ during the early developmental stages. To this end, we generated 11 cDNA libraries derived from expressed sequences tags (ESTs) isolated from three developmentally significant time points in the squid-vibrio system. This approach has been successfully used in the past to identify the set of transcribed genes specific to an organ or tissue of a particular organism (see *e.g.*, [16-20]). The generation of these resources paves the way for an in-depth examination of the influence of symbiosis in host gene expression in the squid-vibrio system.

## Results

**Figure 3:**
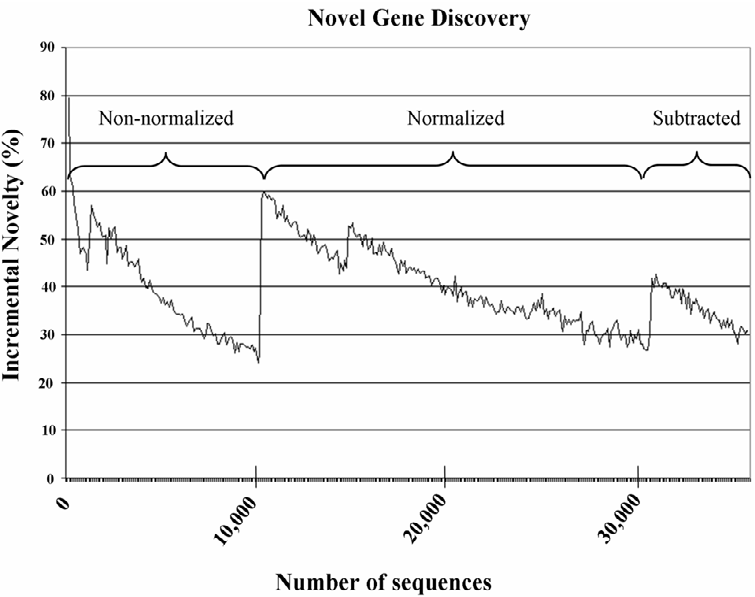
**Novel gene discovery**. Large increases in sequence contributions are evident from each set of libraries, which are illustrated as follows: sequences from 5 non-normalized libraries (approximately the first 10,000 ESTs); sequences from 5 normalized libraries (approximately from 10,000 to 30,000 ESTs); and sequences from pooled, subtracted libraries (after 30,000 ESTs) (Table 1). Novelty rates, calculated as the ratio of the total number of clusters identified over the total number of ESTs generated, were 39%, 54%, and 75%, for the non-normalized, normalized and subtracted libraries, after production of a total of 10,000 (3,920 clusters), 20,190 (10,822 clusters), and 5,171 (3,917 clusters) ESTs, respectively.

A total of 11 cDNA libraries (Table 1) were generated from 5 pools of squid light organ RNA, containing clones with insert sizes ranging from 0.35 to 2.5 kb and an average length of ~1 kb. From these cDNA libraries, sequencing generated a total of 42,564 3' EST sequences with an average sequence length of 692 bp and an average phred value of 39.7. An overall novelty of greater than 45% remained throughout the sequencing phases of the non-normalized, normalized and subtracted libraries (Fig. 3).

Of the 42,564 ESTs generated, 35,421 ESTs satisfied the quality criteria (see Materials and Methods). From the sequences that passed the quality criteria 97% (34,249) contained a polyadenylation tail. Of those sequences with a polyadenylation tail, 63% (21,439) contained a canonical polyadenylation signal sequence (AATAAA or ATTAAA), and another 15% (5,157) contained an alternative polyadenylation signal sequence[21]. In silico comparison of these 42,564 ESTs to sequences from the *V. fischeri *genome showed no sequence similarity, suggesting neither contaminating *V. fischeri *sequence nor evidence for horizontal gene transfer.

These 35,421 ESTs were divided into 13,962 unique clusters using the UIcluster program. One sequence from each cluster was chosen to be a part of a nonredundant set of ESTs. Within this set of clusters, 13,441 contained at least one sequence with a polyadenylation tail and 8,806 of these also contained a polyadenylation signal sequence. Five prime sequences with an average length of 683 bp were generated from these nonredundant representative sequences from each cluster. Of these sequences, 58% (8,067) of 13,962 ESTs contained sufficient overlapping paired 3' and 5' sequences to form contigs with an average sequence length of 1,065 bp. These contigs were used in place of the individual 3' and 5' sequences in further analyses when available. Representative 3' and 5' sequences from the final non-redundant set of ESTs were deposited in the dbEST division of GenBank.

**Figure 4:**
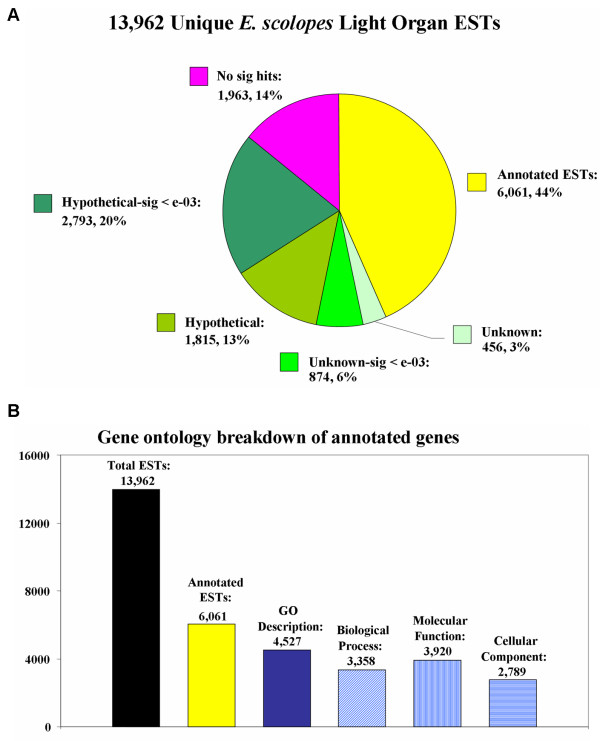
**Annotation of 13,962 unique *E. scolopes *light organ sequences**. A. A pie chart indicating the number of genes and the percent of ESTs annotated as a known protein, unknown protein or a hypothetical protein. All sequences that are annotated as a known protein or as significant (sig) have a BLASTX hit below the E-value threshold of e-03. B. A histogram illustrating the total number of unique *E. scolopes *ESTs, the total number of ESTs annotated as a known protein with an E-value threshold of e-03, the total number of ESTs annotated with at least one category of Gene Ontology (GO) and the number of genes annotated in each of the 3 major GO categories, biological process, molecular function and cellular component.

The BLAST annotation of the nonredundant set (13,962 ESTs) showed that 70% (9,728) had a significant hit to a protein in the NCBI nonredundant protein database with an E-value threshold of e-03 (Fig. 4). Of these significant hits, 62% (6,061) hit an annotated known protein, 29% (2,793) hit a hypothetical protein and 9% (874) hit an unknown protein. Of the remaining 30% without a significant BLAST hit, nearly half, 46% (1,963), may represent novel sequences because there was no hit to the non-redundant database.

Using the DAVID program, GO terms for biological process, molecular function and cellular component were associated with 75% (4,527) of the 6,061 ESTs that hit with an annotated protein in the NCBI database (Fig. 4 and see Additional files 1, 2 and 3). Of these 4,527 ESTs, 78% (3,512) have an associated biological process description, 90% (4,071) have an associated molecular function description, and 65% (2,960) have an associated cellular component description.

Of the 13,962 ESTs, the largest number of ESTs (3,039) was found to be specific to the 48-h symbiotic library and this number is nearly twice the number found to be specific from any other library (1,311 specific to the hatchling library, 1,381 specific to the 12-h aposymbiotic library, 1,314 specific to the 48-h aposymbiotic library, and 1,699 specific to the 12-h symbiotic library). In addition, 29% (4,006) of the 13,962 ESTs are sequences derived from only aposymbiotic libraries (1,311 from hatchling libraries, 1,381 from 12-h aposymbiotic libraries and 1,314 from 48-h aposymbiotic libraries) and 34% (4,738) are derived from only symbiotic libraries (1,699 from 12-h symbiotic libraries and 3,039 from 48-h symbiotic libraries).

Cluster analysis also revealed that 55% (7,639) of 13,962 clusters are singleton clusters (representative of only one EST) and of these, 37% (2,794) are associated with a known protein, 23% (1,720) with a hypothetical protein, and 7% (523) with an unknown protein at an E-value threshold of e-03. Of the singleton clusters, 16% (1,221) do not have a BLAST hit and may be novel. In the set of clusters containing more than 1 EST, 52% (3,271) are associated with a known protein, 17% (1,073) hit with a hypothetical protein, and 6% (351) match with an unknown protein at an E-value threshold of e-03. Of the remaining 45% (6,323) of clusters containing two or more ESTs, 12% (742) do not have a BLAST hit. Although most of these clusters are represented by two or three ESTs, more than a quarter are composed of at least four ESTs with one cluster of 57 ESTs.

The similarity of *E. scolopes *sequences to those of three animals that are commonly studied but distantly related to *E. scolopes*, *Homo sapiens*, *Drosophila melanogaster *and *Caenorhabditis elegans*, were determined using a BLAST-based analysis with an E-value threshold of e-50. The goal of this analysis was to perform a comparative genomic analysis through identification of orthologous genes. This is an alternate strategy which is based on gene content as opposed to a traditional molecular phylogeny analysis which is based on gene sequence. We evaluated several genes using the PHYLIP package (data not shown) with indeterminate results[22]. The results of these analyses indicate the squid ESTs have the greatest number of genes with similarity only to *H. sapiens *genes with 1.30% (342) of the 26,309 annotated human sequences, compared to 0.22% (40) of the 18,289 annotated sequences reported in *D. melanogaster *and 0.03% (6) of the 22,215 annotated sequences reported in *C. elegans *(see Additional file 4). These data show a higher number of squid genes homologous to a deuterostome chordate, *H. sapiens*, than to two members of the Ecdysozoa, the arthropod *D. melanogaster *and the nematode *C. elegans*.

To assess characteristics of the untranslated region and codon usage, a BLAST search was performed between the SwissProt amino acid databases and the assembled contigs. A total of 8% (627) of the 8,067 assembled contigs contained a BLAST hit of at least e-40 with a stop consistent with the carboxy terminus of at least one protein (+/- 5 AA). This same analysis estimated an average 3' UTR length of 227 bp. This estimate is conservative as genes with a UTR longer than the average contig length of 1,065 bp may not have a BLAST hit. These same UTR predictions were used to estimate the GC content in the coding sequence compared to the untranslated sequence. A bias in GC content was observed between the coding and non-coding sequence from 31.5% GC in the predicted untranslated sequence to 42.4% GC content in the predicted coding sequence. Of the nine least frequently used codons six are exclusively composed of G's and C's. These codons were significantly under-used ranging in prevalence from 0.5% – 0.75%. However, the codon GGC, occurs at a frequency of 1.3%, and the codon GCC, occurs at a frequency of 1.6%, and due to the increased prevalence, these two codons are considered significant outliers.

## Discussion

In this report, we characterize 13,962 nonredundant EST sequences generated from eleven cDNA libraries derived from pools of juvenile *E. scolopes *light organs, which were collected at developmentally significant time points with and without colonization by *V. fischeri*. These nonredundant sequences were: 1) characterized, 2) annotated, and 3) analyzed for novelty within each condition.

UniGene sets of over 40,000 unique transcripts have been reported for vertebrate chordates. However, among the invertebrate chordates and other invertebrates, they are most often less than half that size, ranging from approximately 6,000 to 15,000 unique transcripts. Based on clustering analysis, which estimates the number of transcripts present in the organism, the *E. scolopes *nonredundant EST set containing 13,962 clusters is in the same range as that of other invertebrates represented in the UniGene database. It is interesting to note, that the UniGene database reports unique sets as composites of all reported EST sequences, *i.e*., it is not organ specific, thus, the *E. scolopes *nonredundant set is relatively large for an organ specific library and may indicate a substantially larger transcriptome than has previously been reported for other invertebrates. A comparison of the EST clusters derived from the eleven squid light organ libraries showed that the largest number of unique clusters is associated with the 48-h symbiotic condition. This increase in transcriptional diversity is supported by two-dimensional polyacrylamide gel electrophoresis analysis of standing stock protein in the developing squid light organ, which showed that differences in light organ protein profiles in response to *V. fischeri *colonization are not easily detectable until 48 hours post-inoculation [23].

Sequencing of the eleven squid light organ cDNA libraries resulted in ESTs of similar length and quality to sequences generated from previously constructed cDNA libraries [16-18,24]. The predicted 3'-UTR length of 227 bp is shorter than the 411 bp 3'-UTR length found for orthologous human and rat genes [25]. This may be explained, at least in part, by the occurrence of A-rich sequences within the 3' UTRs of a significant fraction of *E. scolopes *transcripts. This hypothesis is based on the fact that 22% of the squid ESTs do not contain a recognizable polyadenylation signal sequence within 30 bases from the 3' end tail, which suggests that such ESTs might result from internal oligo-dT priming during first-strand cDNA synthesis.

BLASTX annotation of these 13,962 sequences identified a number of genes encoding proteins known to be important in the squid light organ for both development and symbiosis, such as: reflectin [26], actin [27], myeloperoxidase [28], aldehyde dehydrogenase [29] and nitric oxide synthase [30]. The identification of genes in the EST database known to play a significant role in the squid light organ serves as a proof-of-concept, suggesting that the additional sequences provided by the construction of the database will be invaluable for the identification of pathways involved in the early development of the squid light organ and its colonization by *V. fischeri*. For example, this EST library has already played an integral role in the discovery of elements of the NF-kB pathway in the host squid [31].

Previous work on the *E. scolopes *– *V. fischeri *system has shown that the light organ is undergoing a rapid morphogenesis in response to signals from both the external environment and colonizing *V. fischeri *in these early hours of the symbiosis (Fig. 2)[13]. The numbers of ESTs in each library as well as Gene Ontology (GO) descriptions of annotated ESTs are reflective of the active state of the organ during these early developmental stages. Specifically, categories overrepresented included those involved with signal transduction, cell membrane, development and morphogenesis. Expression of genes in these categories is not surprising in light of the fact that the host squid and the bacterial symbiont signal to one another through their cell surfaces, an activity that leads to the dramatic remodeling of host tissues. EST sequences often reflect the function of tissue from which the cDNA library was derived. To determine if the patterns of genes expressed in these cDNA libraries are unique to the squid light organ, we compared our results with that of the nonredundant EST database derived from the developing rat heart [17]. The squid light organ database is dominated by signal transduction (67%) and membrane-associated proteins (49%), whereas the database of the rat heart is dominated by cell growth and maintenance (63%) and intracellular activities (70%). Thus, in a broad sense, the content of these databases appear to mirror the physiological state of these organs.

## Conclusion

This EST library is the first effort to generate a bioinformatics tool for the host side of the squid-vibrio association. This tool will allow us use this powerful binary model to ask specific questions to elucidate basic host responses to bacterial colonization in vivo, offering a complement to the more complex associations of vertebrates and the microbiota associated with their mucosa. Finally, the creation of a light organ-specific spotted microarray using this EST library will pave the way for the study of broad scale changes in host gene expression that underlie the dramatic developmental program of this symbiosis.

## Methods

### Animal maintenance and tissue preparation

Adult *E. scolopes *and egg clutches produced by female adults were maintained at the Kewalo Marine Laboratory as previously described [32]. Approximately 1,500 light organs were collected in RNALater (Ambion) for the eventual production of cDNA libraries from juvenile squid in five conditions: hatchling, 12-h aposymbiotic, 12-h symbiotic, 48-h aposymbiotic, and 48-h symbiotic animals. All animals were hatched into Hawaiian offshore seawater (HOSW), which does not contain enough *V. fischeri *cells to result in light organ colonization, but does contain other bacteria (at 10^5^-10^6 ^cells/ml) that naturally occur in the waters off of the Hawaiian island of Oahu. All light organs were dissected from animals, which had been anesthetized for ~2 min in 2% ethanol and HSOW, within 1 h before and after the time point specified for each condition, except for the hatchling condition, for which all dissections occurred within 1 h post-hatching.

### RNA isolation

Total RNA was extracted from light organs in RNALater (Ambion) using Trizol reagent (Gibco BRL, Rockville, MD). Total RNA was quantitated by spectrophotometry and the quality was determined by 2% formaldehyde-agarose gel electrophoresis. Poly(A)^+ ^RNA was isolated from total RNA samples using oligo-(dT)-cellulose chromatography.

The method used for the construction of directionally cloned cDNA libraries [33,34] includes a column chromatography step that is aimed at eliminating unwanted DNA fragments (primers and adaptors) and short cDNAs (*e.g.*, those consisting exclusively of poly(A) tail). Although aware of the possibility of excluding cDNAs derived from genuine short transcripts, this step has proven important to minimize generation of nuisance ESTs in large-scale sequencing projects.

### cDNA libraries

DNase-treated poly-(A)^+ ^samples were used to create 11 cDNA libraries: 5 start (non-normalized), 5 normalized and 1 subtracted. For each library, cDNA was primed with the following oligo (dT) primer, [TGTTACCATTCTGATGTTGGAGCGGCCGC-N[6–10]-T[18]]. Each primer contained a NotI restriction site for directional cloning and one of 11 unique library tags of 6 to 10 nucleotides (N [6–10]), which identifies the condition of origin (Table 1) [35]. Double-stranded cDNA was ligated to EcoRI adaptors [5'-AATTGGCACGAGG-3', 3'-GCCGTGCTCC-5'], digested with NotI, and directionally cloned into the phagemid vector pT7T3-Pac as described in [17]. Each library comprised several times more recombinants than the expected number of transcripts from the RNA populations utilized, and thus can be treated as if they had the same number of primary recombinants.

### Sequencing, analysis and clustering

Di-deoxy terminator sequencing was performed from the 3' end of the cDNA clones using M13 forward (5'-GTTTTCCCAGTCAC-3') primers in a 96-well format via cycle sequencing with dRhodamine dye terminator chemistry (Applied Biosystems, Foster City, CA). After thermal cycling, sequencing reactions were processed and analyzed on an ABI-377 or an ABI-3700 capillary sequencer as described in [36].

After data capture on the ABI sequencers, the chromatographs are transferred to a centralized server. From there, the sequences were processed as outlined below and placed into our local file-system. Nucleotide sequences and per-base quality values were extracted from the ABI-generated chromatograph files (SCF files) using the Phred-base-calling program and evaluated for 3 features: 1) overall sequence quality (Phred q-score >25); 2) percent of sequence (in nt) over q20 > 50%, and 3) the quality-trimmed EST insert length of more than 100 bp [37,38].

ESTprep [39] and RepeatMasker (Smit and Green, unpublished data) programs were used to assess the presence of the following EST features: vector cloning site, restriction site, polyadenylation tail and signal sequence, library tag, and potential contaminating sequences from *Escherichia coli*, *V. fischeri *and vector as described in [17,40]. Local clustering of the ESTs was performed using the sequence-based clustering program UIcluster [41], allowing matches based on both the forward and reverse complements. A representative sequence was selected from each cluster based on best sequence quality, as described above, resulting in a unigene set containing 13,962 ESTs. Each of the cDNA clones in the unigene set was sequenced from the 5' end using the same protocol described above with the exception of using the M13 reverse primer (5'-AGCGGATAACAATTTCACACAGGA-3') instead of the M13 forward primer. The 3' and 5' sequences were assembled into a consensus contig using default settings of the Phred program when sufficient quality sequence existed. 3' and 5' sequences generated as a part of this research were submitted to dbEST division of GenBank at National Center for Biotechnology Information (NCBI) under accession numbers ranging from Genbank:DW251302 to Genbank:DW286722[42].

### Sequence annotation

Each of the 13,962 ESTs were compared against the six nonredundant peptide sequence databases (GenBank CDS translations, RefSeq Proteins, PDB, SwissProt, PIR, and PRF) available on NCBI using a BLAST-based analysis program (BLASTX), which translates each EST sequence in six reading frames prior to each query. BLAST analyses against peptide sequences provide the largest number of hits in comparison to nucleotide sequences because peptide analyses allow for nucleotide sequence divergence among species. Default BLASTX parameters were used including a maximum expectation (E)-value of 10. Because of the low abundance of sequence data from organisms in the sub-kingdom containing mollusks (*i.e*., Lophotrochozoa) available in NCBI's database, we chose a high E-value threshold to reveal as many potential matches to our squid EST sequences as possible, *i.e*., sequence annotation was performed using an E-value threshold of e-03 to determine significance. Sequences were first annotated based on a significant hit to an annotated protein. If no hit to an annotated protein occurred, then sequences were annotated as a 'significant' or a 'non-significant' 'unknown' (match to a predicted protein sequence from a cDNA or other nondescriptive term) or 'hypothetical' (match to a hypothetical protein). Finally, if the result of the BLAST analysis showed no significant hits to any protein in the NCBI nonredundant database, sequences were annotated as 'no significant hit'. The gene name and a unique identifier (*i.e*., GI number, Accession Number, Entrez gene ID, Genepept accession) were collected from the NCBI web site for each chosen BLAST hit. Putative functions and product information, such as Gene Ontology (GO) for each unique identifier matching an annotated protein was determined with the Database for Annotation Visualization and Integrated Discovery (DAVID) [43]. Annotated ESTs were categorized based on the terms in each of the standard GO categories, specifically biological process (BP), molecular function (MF) and cellular component (CC).

## Authors' contributions

MBS, TLC and MJM-F are the Principal Investigators who were involved in the design of this study. MBS designed and coordinated all aspects of the project. MJM-F conceived the study, coordinated tissue collection and obtained funding for research reported in this manuscript. TLC coordinated the creation of the EST databases. MFB coordinated the creation of the cDNA libraries and the EST databases. CKC, JVT, MSG, WJC-G, AC, TD, BJ, TAK, CY and JWR collected tissue for the creation of these libraries. JJS and JLK participated in isolating RNA for the creation of these libraries. In addition, CKC participated in the design and annotation of the EST database, performed gene ontology analyses and drafted the manuscript. ME and SW created the cDNA libraries. KC, TK and CS sequenced the cDNA libraries. TES participated and coordinated the creation of the EST database. BB created the EST database. DT annotated the sequences in the EST libraries. All authors read and approved the final manuscript.

## Supplementary Material

Additional File 1 **Figure S1 – GO Biological Process (BP)**. A. A histogram illustrating the breakdown of GO/BP annotated ESTs in each of BP first level subcategories. B. *Top *refers to the distribution of ESTs in 3 first level categories, cellular process (CP), physiological process (PP) and development (D). *Bottom *refers to the distribution of ESTs in three lower level categories from CP, signal transduction/cell surface receptor linked signal transduction, PP, response to stimulus and D, morphogenesis. Click here for file

Additional File 2 **Figure S2 – GO Molecular Function (MF)**. A. A histogram illustrating the breakdown of GO/MF annotated ESTs in each of MF first level subcategories. B. Refers to the distribution of ESTs in 3 first level categories, binding, catalytic and structural. Click here for file

Additional File 3 **Figure S3 – GO Cellular Component (CC)**. A. A histogram illustrating the breakdown of GO/CC annotated ESTs in each of CC first level subcategories. B. *Left *refers to the distribution of ESTs in one of the first level categories, cell. *Middle and right *refer to the distribution of ESTs in two lower level categories from cell, membrane and intracellular. Click here for file

Additional File 4 **Figure S4 – Venn diagram of commonly annotated sequences in *E. scolopes*, *H. sapiens*, *D. melanogaster*, and *C. elegans***. Annotation of *E. scolopes *nonredundant sequences using an E-value threshold of e-50. The numbers of homologous genes identified are represented in each section of the Venn diagram and the total number of sequences used in this comparison is listed under the name of each organism. Click here for file

## Figures and Tables

**Table 1:** EST library descriptions.

**Library**	**Number of ESTs**	**Description**
UI-S-GB0	1,174	Aposymbiotic; 0 hours
UI-S-GB1	4,481	Aposymbiotic; 0 hours; normalized
UI-S-GG0	964	Aposymbiotic; 12 hours
UI-S-GG1	4,292	Aposymbiotic; 12 hours; normalized
UI-S-GN0	1,552	Symbiotic; 12 hours
UI-S-GN1	5,187	Symbiotic; 12 hours; normalized
UI-S-GS0	1,839	Aposymbiotic; 48 hours
UI-S-GS1	4,151	Aposymbiotic; 48 hours; normalized
UI-S-GU0	4,472	Symbiotic; 48 hours
UI-S-GU1	2,079	Symbiotic; 48 hours; normalized
UI-S-HH0	5,171	Pooled, subtracted library from UI-S-GB1, -GG1, -GN1, -GS1, -GU1
