# Supplementary figures and images for: An annotated cDNA library of juvenile Euprymna scolopes with and without colonization by the symbiont Vibrio fischeri
Source: BMC Genomics. 2006 Jun 16;7:154. doi: 10.1186/1471-2164-7-154 (PMC1574308; doi:10.1186/1471-2164-7-154)

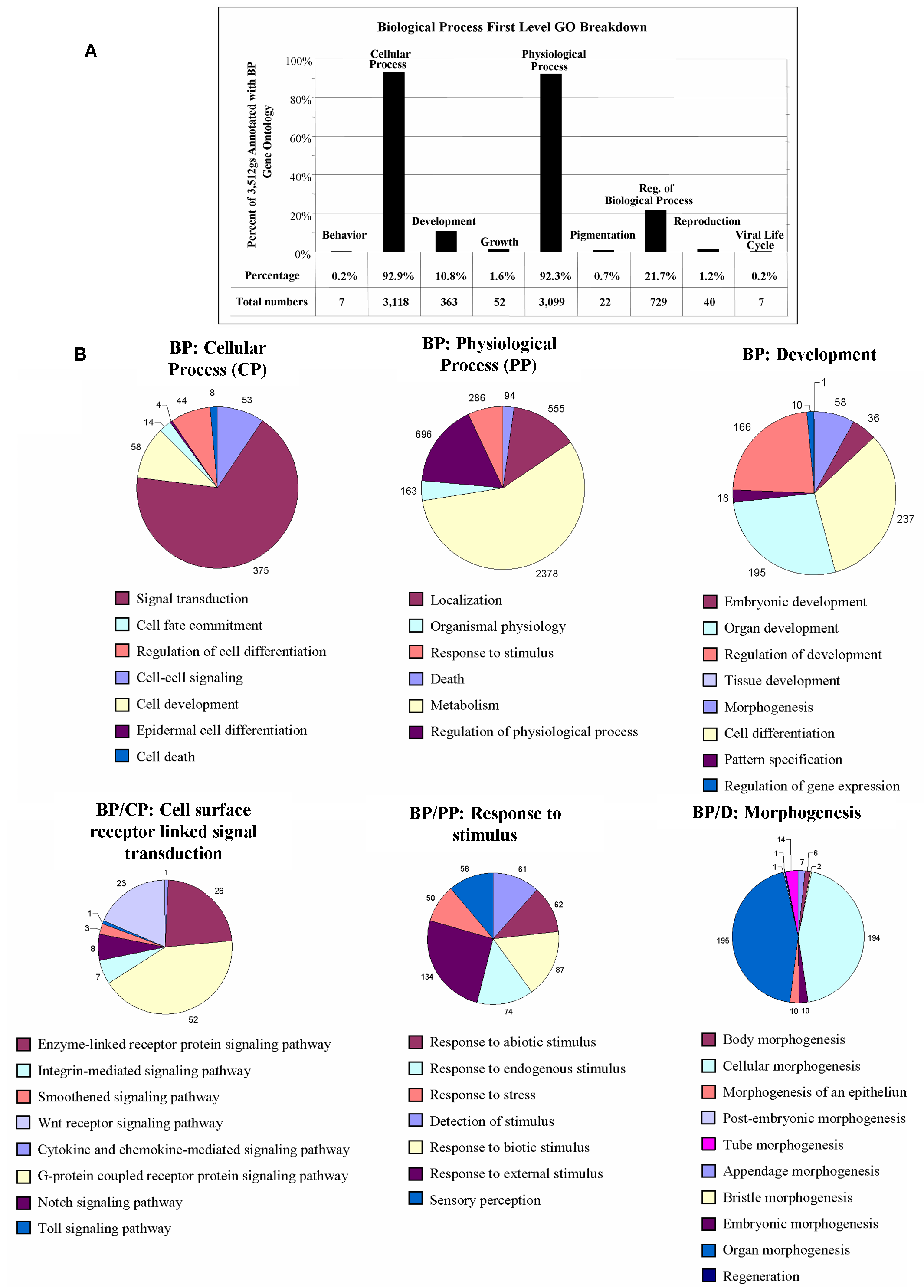

Supplement: Additional File 1 — Figure S1 – GO Biological Process (BP). A. A histogram illustrating the breakdown of GO/BP annotated ESTs in each of BP first level subcategories. B. Top refers to the distribution of ESTs in 3 first level categories, cellular process (CP), physiological process (PP) and development (D). Bottom refers to the distribution of ESTs in three lower level categories from CP, signal transduction/cell surface receptor linked signal transduction, PP, response to stimulus and D, morphogenesis. [file 1471-2164-7-154-S1.tiff]

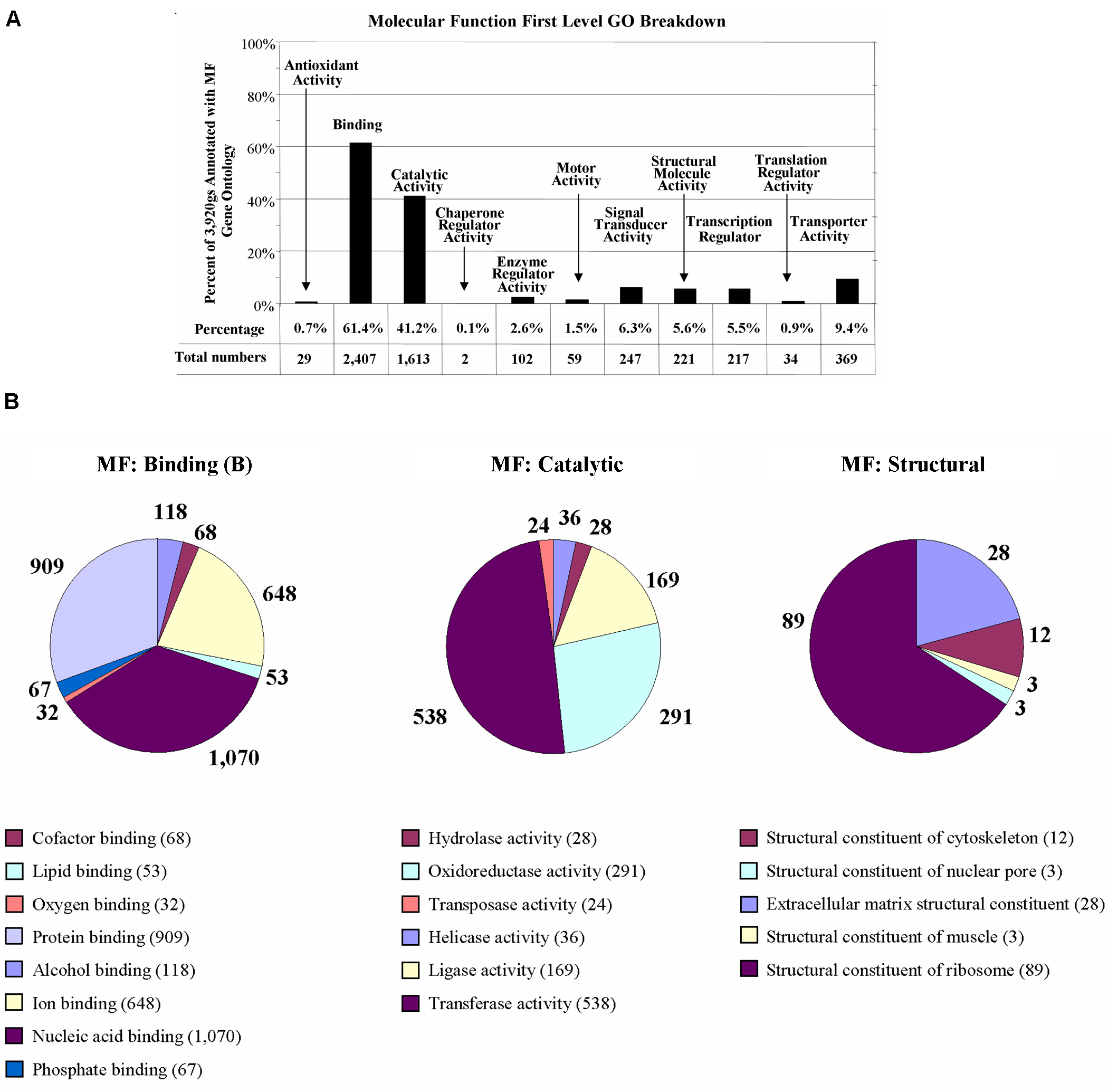

Supplement: Additional File 2 — Figure S2 – GO Molecular Function (MF). A. A histogram illustrating the breakdown of GO/MF annotated ESTs in each of MF first level subcategories. B. Refers to the distribution of ESTs in 3 first level categories, binding, catalytic and structural. [file 1471-2164-7-154-S2.tiff]

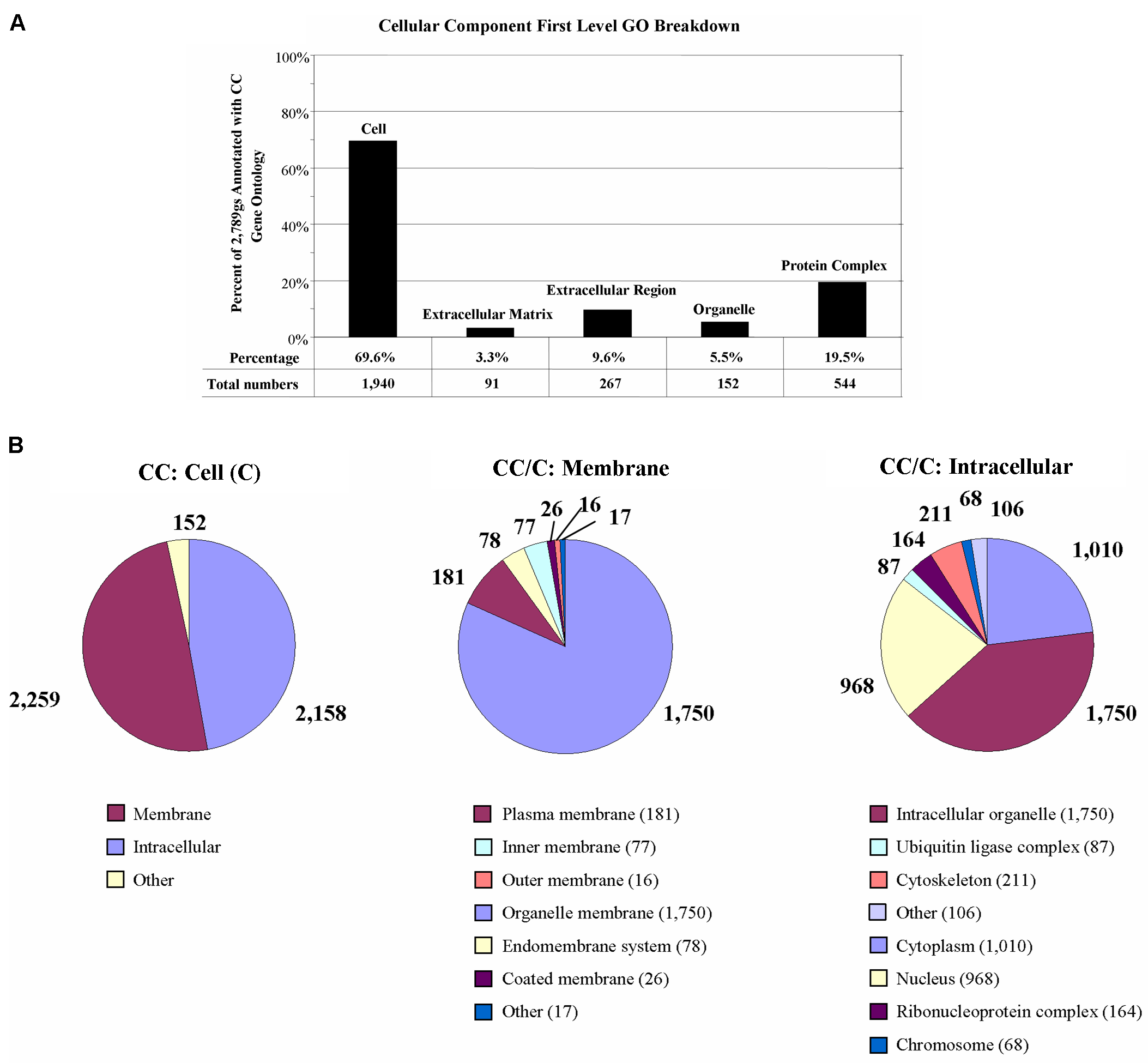

Supplement: Additional File 3 — Figure S3 – GO Cellular Component (CC). A. A histogram illustrating the breakdown of GO/CC annotated ESTs in each of CC first level subcategories. B. Left refers to the distribution of ESTs in one of the first level categories, cell. Middle and right refer to the distribution of ESTs in two lower level categories from cell, membrane and intracellular. [file 1471-2164-7-154-S3.tiff]

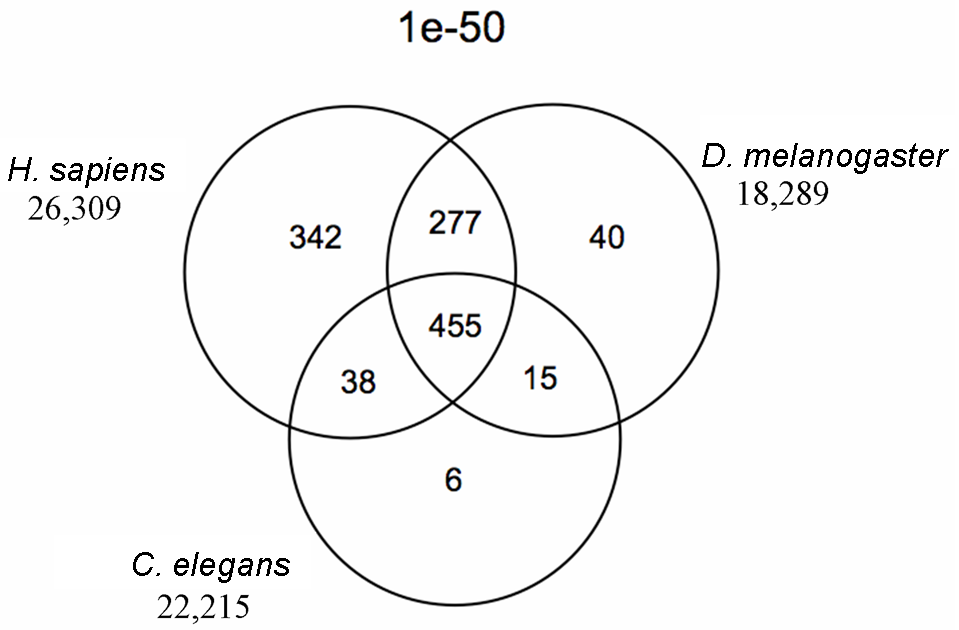

Supplement: Additional File 4 — Figure S4 – Venn diagram of commonly annotated sequences in E. scolopes, H. sapiens, D. melanogaster, and C. elegans. Annotation of E. scolopes nonredundant sequences using an E-value threshold of e-50. The numbers of homologous genes identified are represented in each section of the Venn diagram and the total number of sequences used in this comparison is listed under the name of each organism. [file 1471-2164-7-154-S4.tiff]
